# Characterization of serious adverse drug reactions as cause of emergency department visit in children: a 5-years active pharmacovigilance study

**DOI:** 10.1186/s40360-018-0207-4

**Published:** 2018-04-16

**Authors:** Niccolò Lombardi, Giada Crescioli, Alessandra Bettiol, Ettore Marconi, Antonio Vitiello, Roberto Bonaiuti, Anna Maria Calvani, Stefano Masi, Ersilia Lucenteforte, Alessandro Mugelli, Lisa Giovannelli, Alfredo Vannacci

**Affiliations:** 1 Department of Neurosciences, Psychology, Drug Research and Child Health, Section of Pharmacology and Toxicology, University of Florence, viale G. Pieraccini, 6, 50139 Florence, Italy; 2 Department of Pharmacy, Anna Meyer Children’s University Hospital, viale G. Pieraccini, 24, 50139 Florence, Italy; 3 Department of Emergency Medicine, Anna Meyer Children’s University Hospital, viale G. Pieraccini, 24, 50139 Florence, Italy

**Keywords:** Pediatrics, Adverse events, Safety, Observational study, Preventability, Drug interactions

## Abstract

**Background:**

To describe frequency, preventability and seriousness of adverse drug reactions (ADRs) in children as cause of emergency department (ED) admission and to evaluate the association between specific factors and the reporting of ADRs.

**Methods:**

A retrospective analysis based on reports of suspected ADRs collected between January 1st, 2012 and December 31st, 2016 in the ED of Meyer Children’s Hospital (Italy). Demographics, clinical status, suspected drugs, ADR description, and its degree of seriousness were collected. Logistic regression was used to estimate the reporting odds ratios (RORs) with 95% confidence intervals (CIs) of potential predictors of ADR seriousness.

**Results:**

Within 5 years, we observed 834 ADRs (1100 drug-ADR pairs), of whom 239 were serious; of them, 224 led to hospitalization. Patients were mostly treated with one drug. Among patients treated with more than one drug, 78 ADRs presented a potential interaction. The most frequently reported ADRs involved gastrointestinal system. The most frequently reported medication class was antinfectives. Risk of serious ADR was significantly lower in children and infants compared to adolescents (ROR 0.41 [95% CI: 0.27–0.61] and 0.47 [0.32–0.71], respectively), and it was significantly increased in subjects exposed to more than one drug (ROR 1.87 [1.33–2.62] and 3.01 [2.07–4.37] for subjects exposed to 2 and 3 or more drugs, respectively). Gender, interactions and off-label drug use did not influence the risk of serious ADRs.

**Conclusion:**

Active surveillance in pharmacovigilance might represent the best strategy to estimate and characterize the clinical burden of ADRs in children.

**Electronic supplementary material:**

The online version of this article (10.1186/s40360-018-0207-4) contains supplementary material, which is available to authorized users.

## Background

Adverse drug reactions (ADRs) are the most common cause of iatrogenic harm in health care and have recently received attention in national patient safety initiatives [1]. Moreover, ADRs are a significant cause of emergency department (ED) visits [2].

Most studies on hospital admissions related to medication use focus on adult patients [3–8]. Fortunately, the burden of ADR-related ED admissions in children seems to be lower than observed in adults (5%–25% of all ED admissions) [3, 9], ranging from 0.4% to 10.3% of all children with a pooled estimate of 2.9% [10].

In this context, risk factors for ADRs in children are still poorly characterized. In fact, pharmacotherapy in children differs from adults, thus the spectrum of ADRs in children may differ as well [11], and certain subgroups are at higher risk, such as children exposed to cancer chemotherapy [12]. Furthermore, children are more often exposed to off-label medications, that potentially represent a greater risk for ADRs [13]. Because part of these medication-related events may be preventable [14], more knowledge on occurrence and content of ADRs in pediatric setting is necessary, with efforts focused on reducing medication errors and inappropriate prescribing [1].

EDs are an essential part of health care systems, serving as an interface between hospitals and communities, and could constitute the most important source of information about the frequency, seriousness, and economic burden of ADRs [15, 16].

Preventing ADRs and their related ED admissions in outpatients remains a public health and a patient safety challenge. Aims of this study are to describe frequency, preventability and seriousness of ADRs in children, and to evaluate the association between specific factors and the reporting of ADRs, by means of a 5-years long active pharmacovigilance study in ED.

## Methods

This is a retrospective study performed on reports of suspected ADRs collected between January 1st, 2012 and December 31st, 2016 in the ED of Anna Meyer Children’s University Hospital of Florence (Italy), as part of the study “Epidemiological Monitoring of Adverse Drug Reactions in Emergency Department” (MEREAFaPS), an on-going multicenter Italian active pharmacovigilance project. We analyzed all suspected ADRs reported from patients having at least one drug- or vaccine-related event.

All suspected ADRs leading to ED admission were identified from ED clinical charts and hospitalization data from the hospital discharge database. For each ADR report, we recorded demographic characteristics (age, gender, ethnic group), and patient clinical status on ED admission. Anatomical Therapeutic Chemical (ATC) classification system was used to code ongoing therapy (suspect and concomitant medications, administration route, therapy duration, and dosages). We also recorded all therapeutic indications of suspected drugs. The description of the ADR according to diagnosis and symptoms, was coded using the Medical Dictionary for Regulatory Activities (MedDRA) and organized by System Organ Class (SOC) [17]. ADR seriousness was classified according to the World Health Organization (WHO) criteria [17] as fatal, life-threatening, or requiring hospitalization of the patient, or causing serious/permanent disability.

The primary outcome was the frequency of total and serious ADRs leading to ED admission. The most frequently reported SOC and ATC classes were also evaluated. For each drug-ADR pair, causality and preventability (categorized as definitely or probably preventable, or not preventable) were assessed using the Naranjo [18] scale and Schumock and Thornton [19] algorithm, respectively. For the causality assessment of vaccine-related ADRs, the specific WHO [20, 21] algorithm was used. Data on medications were also analysed for drug-drug interactions (DDIs) by using the drug interaction software Micromedex Drug-REAX System (Thomson Reuters Healthcare Inc., Greenwood Village, Colorado, United States), available online with restricted access [22, 23].

The specific factors associated with the reporting of ADRs, such as age classes, sex, number of suspected drugs, DDIs and on-label vs off-label use were also analysed. The off-label use in children was defined as the use of a drug already covered by a Marketing Authorisation, in an unapproved way in terms of therapeutic indication, posology, formulation and route of administration [24].

Descriptive statistics was used to summarize data. Categorical data were reported as frequencies and percentages, whereas continuous data were reported as means with standard errors. Univariate and multivariate logistic regression were used to estimate the reporting odds ratios (RORs) with 95% confidence intervals (CIs) of potential predictors of ADR seriousness. ROR values for each variable were reported both as crude values and adjusted for all the other considered variables. All results were considered to be statistically significant at *p < 0.05*. Data management and statistical analysis were carried out using STATA 14.

Tuscany approved the present study with the Notification number 1225 - December 21, 2009. The local institutional ethics committee of the coordinating center (*Comitato Etico di Area Vasta Nord “CEAVNO” per la Sperimentazione Clinica, Azienda Ospedaliero-Universitaria Pisana*) approved the study according to the legal requirements concerning observational studies (*Study number 3055/2010, Protocol number 45288 - August 6, 2014*).

## Results

During the 5-year study period, a total of 221,528 ED admissions was evaluated; of them, 492 were ADR-related, with an overall rate of 2.2 per 1000 ED admissions. Table 1 shows data of pediatric patients who experienced an ADR-related ED admission. We observed 834 ADRs, of whom 239 were serious (28.66%) and, of them, 224 led to hospitalization. No ADR led to death. Most of ADRs occurred in Caucasians (90.65%), with a mean patients’ age (± standard error) of 62.53 ± 2.09 months. The male/female rate of ADRs was 1.17, i.e. comparable to the all-cause ED admission rate observed in Meyer Children’s University Hospital. A total of 483 ADRs (57.91%) had an improvement and 48 (5.76%) had a complete resolution. For 285 ADRs (34.17%) data on the outcome were not available. Overall, patients were mostly treated with only one drug at time of ADR occurrence. Among patients treated with two or more drugs, 78 ADRs (20.21%) presented a potential DDI, which was severe in 57 cases (14.77%). The total number of drug-ADR pairs was 1100. Of them, 711 drug-ADR pairs were related to non-vaccines drugs; considering causality assessment, 82 were certain, 310 probable, 301 possible and 18 in doubt. On the other hand, 389 (35.36%) were related to vaccines; in particular, considering the evaluation of causality assessment, 333 were considered as associable, 50 as undefined and 6 were not associable.

**Table 1:** Characteristics of pediatric patients admitted to ED for ADR

	Tot ADRs N (% out of 834)
N involved patients	492
N ADRs/patients	
1	256 (52.03)
2	155 (31.50)
3+	81 (16.46)
Age	
Mean ± standard error, months	62.53 ± 2.09
Age classes	
New-borns (< 1 month)	7 (0.84)
Infants (1–23 months)	332 (39.81)
Children (24–143 months)	368 (44.12)
Adolescents (144–192 months)	127 (15.23)
Sex	
Male	449 (53.48)
Female	385 (46.16)
Ethnic group	
Caucasian	756 (90.65)
Others	25 (3.00)
Not available	53 (6.35)
Tot number of drugs	
1	448 (53.72)
2	255 (30.58)
3+	131 (15.71)
Seriousness	
Hospitalization	224 (26.86)
Congenital abnormalities/ Deficit	1 (0.12)
Other clinically relevant conditions	14 (1.68)
Outcome	
Complete resolution	48 (5.76)
Improvement	483 (57.91)
Invariant situation/worsening	18 (2.16)
Not available	285 (34.17)
Drug-drug interactions^a^	
No	308 (79.79)
Yes	78 (20.21)
Mild	4 (1.04)
Moderate	17 (4.40)
Severe	57 (14.77)
	Tot drug ADR-pairs N (% out of 1100)
Drugs (vaccines excluded)	711 (64.64)
Causality	
In doubt	18
Possible	301
Probable	310
Certain	82
Vaccines	389 (35.36)
Causality	
Not associable	6
Undefined	50
Associable	333

^a^Percentage calculated on the total number of ADRs occurred in patients with more than one drug

Table 2 reported the distribution of ADRs according to SOC classification. The most frequently reported SOCs were: gastrointestinal disorders (27.34%), followed by general disorders and administration site conditions (21.46%), skin and subcutaneous tissue disorders (16.19%), nervous system disorders (12.95%) and metabolism and nutrition disorders (3.60%). The majority of ADRs for each SOC were non-serious.

**Table 2:** Distribution of ADRs according to SOC classification

	Tot ADRs N (% out of 834)	Serious ADRs N (% out of corresponding SOC)
SOC		
Gastrointestinal disorders	228 (27.34)	*68 (29.82)*
General disorders and administration site conditions	179 (21.46)	*41 (23.56)*
Skin and subcutaneous tissue disorders	135 (16.19)	*17 (12.59)*
Nervous system disorders	108 (12.95)	*45 (41.67)*
Metabolism and nutrition disorders	30 (3.60)	*11 (36.67)*
Respiratory, thoracic and mediastinal disorders	27 (3.24)	*8 (29.63)*
Musculoskeletal and connective tissue disorders	27 (3.24)	*11(40.74)*
Psychiatric disorders	18 (2.16)	*7 (38.89)*
Eye disorders	13 (1.56)	*5 (38.46)*
Cardiac disorders	12 (1.44)	*5 (41.67)*
Ear and labyrinth disorders	10 (1.20)	*1 (10)*
Investigations	8 (0.96)	*3 (37.50)*
Infections and infestations	8 (0.96)	*1 (12.50)*
Vascular disorders	7 (0.84)	*2 (28.57)*
Injury, poisoning and procedural complications	7 (0.84)	*4 (57.14)*
Blood and lymphatic system disorders	5 (0.60)	*3 (60.00)*
Reproductive system and breast disorders	4 (0.48)	*0 (0.00)*
Renal and urinary disorders	4 (0.48)	*3 (75.00)*
Immune system disorders	3 (0.36)	*3 (100.00)*
Surgical and medical procedures	1(0.12)	*1 (100.00)*

The drug-ADR pairs (stratified by ATC classes, level I) most commonly involved in ED admissions, overall and stratified according to ADRs seriousness, preventability and off-label use are listed in Table 3. Out of 1100 drug-ADR pairs, 711 were not related to vaccines; of them more than 30% were serious, 24% preventable and 8.7% related to off-label use. The most frequently reported ATC class was antinfectives for systemic use (ATC class J, excluded J07*) accounting for 288 drug-ADR pairs (40.51%). Of them, 23.96% were serious, 24.19% were preventable and 0.69% were related to off-label use. The second most frequently reported ATC class was nervous system medication (ATC class N), accounting for 168 drug-ADR pairs (23.63%), of whom 48.21, 48.81 and 10.71% were serious, preventable and related to off-label use, respectively. All other ATC classes accounted for 8.30 to 0.56% of drug-ADR pairs.

**Table 3:** Distribution of drug-ADR pairs according to ATC classification (level I), overall and stratified according to seriousness, preventability and off-label use

	Tot drug-ADR pairs N (% out of 1100)	Seriousness N (% out of ATC level I)	Preventability N (% out of ATC level I)	Off-label use N (% out of ATC level I)
Related to Vaccines	389 (35.36)	*109 (28.02)*	*27 (6.94)*	*–*
Not related to Vaccines (ATC level I)	711 (64.64)	*226 (31.79)*	*172 (24.19)*	*62 (8.72)*
J - Antinfectives for systemic use (excluded vaccines - J07)	288 (40.51)	*69 (23.96)*	*15 (5.21)*	*2 (0.69)*
N - Nervous system	168 (23.63)	*81 (48.21)*	*82 (48.81)*	*18 (10.71)*
A - Alimentary tract and metabolism	59 (8.30)	*16 (27.12)*	*25 (42.37)*	*3 (5.08)*
M - Musculoskeletal system	53 (7.45)	*18 (33.96)*	*9 (16.98)*	*0 (0.00)*
C - Cardiovascular system	39 (5.49)	*17 (43.59)*	*22 (56.41)*	*18 (46.15)*
H - Systemic hormonal preparations	31 (4.36)	*7 (22.58)*	*6 (19.35)*	*5 (16.13)*
R - Respiratory system	27 (3.80)	*3 (11.11)*	*2 (7.41)*	*3 (11.11)*
L - Antineoplastic and immunomodulating agents	13 (1.83)	*4 (30.77)*	*2 (15.38)*	*2 (15.38)*
B- Blood and blood-forming organs	11 (1.55)	*6 (54.55)*	*3 (27.27)*	*5 (45.45)*
D - Dermatologicals	10 (1.41)	*3 (30.00)*	*1 (10.00)*	*0 (0.00)*
G - Genitourinary system and sex hormones	4 (0.56)	*2 (50.00)*	*4 (100.00)*	*4 (100.00)*
P - Antiparasitic products	4 (0.56)	*0 (0.00)*	*0 (0.00)*	*0 (0.00)*
S - Sensory organs	4 (0.56)	*0 (0.00)*	*1 (25.00)*	*2 (50.00)*

The most frequent suspected active principles (APs) among antinfectives for systemic use agents and nervous system medications are reported in Additional file 1: Table S1.

Out of 1100 drug-ADR pairs, 389 were related to vaccines (35.36%); of them, 28% and 6.9% were serious and preventable, respectively (Table 3). The most frequent suspected vaccines were Meningococcus B, multicomponent vaccine (*n* = 67, of whom 32.84% serious), Diphtheria-haemophilus influenzae B-pertussis-poliomyelitis-tetanus-hepatitis B (*n* = 57, of whom 45.61% serious), and Pneumococcus, purified polysaccharides antigen conjugated (*n* = 55, of whom 32.73% serious) (Additional file 2: Table S2**)**.

Table 4 reports the distribution of serious and non-serious drug-ADR pairs and the potential associations between serious ADR risk and age, gender, total number of pharmacological treatments and presence of DDIs. On-label and off-label use and relative associations were also evaluated. Seriousness of ADRs significantly differed among age classes and according to the total number of drugs administered (*p < 0.001*). Risk of having an ADR reported as serious was significantly lower in children and infants compared to adolescents (adjusted ROR of 0.41 [95% CI: 0.27–0.61] and 0.47 [0.32–0.71], respectively). Risk of serious ADRs was significantly increased in subjects exposed to more than one drug (adjusted ROR of 1.87 [1.33–2.62] and 3.01 [2.07–4.37] for subjects exposed to 2 and 3 or more drugs, respectively) compared to subjects exposed to only one drug. On the other hand, the gender, the presence of DDIs and the on-label or off-label drug use did not influence the risk of having an ADR reported as serious.

**Table 4:** Distribution of serious and non-serious drug-ADR pairs, and association between serious ADR risk and different factors, expressed as Reporting Odds Ratio (ROR)

	Non-serious drug-ADR pairs N (% out of 765)	Serious drug-ADRs pairs N (% out of 335)	*p*-value	Adjusted ROR of serious ADR [95% CI]	*p*-value
Age classes (FDA classification)					
Adolescents (144–192 months)	86 (11.24)	72 (21.49)	< 0.001	Ref	
Children (24–143 months)	335 (43.79)	123 (36.72)		0.41 [0.27–0.61]	< 0.001
Infants (1–23 months)	336 (43.92)	137 (40.90)		0.47 [0.32–0.71]	0.001
New-borns (< 1 month)	8 (1.05)	3 (0.90)		0.42 [0.10–1.70]	*0.224*
Sex					
Male	353 (46.14)	144 (42.99)	*0.333*	Ref.	
Female	412 (53.86)	191 (57.01)		0.77 [0.59–1.02]	*0.072*
Tot number of drugs					
1	347 (45.36)	101 (30.15)	< 0.001	Ref.	
2	276 (36.08)	128 (38.21)		1.87 [1.33–2.62]	< 0.001
3+	142 (18.56)	106 (31.64)		3.01 [2.07–4.37]	< 0.001
Drug-drug interactions					
No	268 (64.11)	165 (70.51)	*0.097*		
Yes	150 (35.89)	69 (29.49)		0.77 [0.54–1.11]	*0.166*
Off-label vs on-label use^a^					
On-Label	447 (92.36)	201 (88.94)	*0.133*	Ref.	
Off-label	37 (7.64)	25 (11.06)		1.56 [0.90–2.73]	*0.115*

Adjusted ROR values are shown along with the respective 95% Confidence Intervals

^a^Calculated only on non-vaccine medications

## Discussion

This study aimed to explore the frequency, preventability and seriousness of ADRs observed over a 5-year period in a single pediatric center in Italy, and to our knowledge it is the first study focused on serious ADRs with the aim of addressing the factors associated with the reporting of this kind of ADRs, in an Italian clinical pediatric setting.

In the present analysis, the rate of ADR-related ED admissions was 2.2 per 1000 admissions, notably lower than what is generally reported in literature (0.4% to 10.3% of all children, pooled estimate of 2.9%) [10]. In fact in a large systematic review [10], the rates of ADRs causing hospital admission ranged from 0.4% to 10.3% of all children (pooled estimate of 2.9% (2.6%, 3.1%)). This can be explained by the fact that we calculated the actual rate of ADRs on all ED admissions, including also those not related to drug utilization. These findings are in keeping with what was reported in another Italian study [25] that showed similar results in terms of ATC classes and SOCs most frequently associated with ADRs in children. According to Rosafio et al. (2017) [25], the medication classes most frequently implicated were anti-infective drugs for systemic use (28.9%) and central nervous system agents (22.3%). The three most common symptom manifestations were dermatologic conditions (46.1%), general disorder and administration site conditions (29.7%) and gastrointestinal symptoms (16.0%).

Among the ADR reports collected in our study, “Antinfectives for systemic use” were the most common drugs, in agreement with a major national ADRs overview conducted in Italy in the last decade [16, 26]. In particular, amoxicillin and amoxicillin/clavulanate were the most frequently involved APs. This evidence could be attributable to the fact that in Tuscany amoxicillin and amoxicillin/clavulanate are generally the most prescribed antibiotics in the pediatric population (14.06 and 23.08 defined daily dose/1000 inhabitants per day, respectively) [27]. The most reported SOC in the present study was “Gastrointestinal disorders”, whereas in literature “Skin and subcutaneous tissue disorders” is generally the first SOC to be reported in this population subset [28]. This discrepancy could be related to the fact that dermatological ADRs are generally less serious than gastrointestinal ones, and therefore could be mainly managed by out-of-hospital healthcare professionals rather that in EDs.

Regarding vaccine-related ED admission, this study showed a higher number of drug-ADR pairs for “Meningococcus B multicomponent vaccine” than reported by Rosafio et al. [25]. This data are not surprising given the peculiar Tuscan scenery of meningococcal B disease high incidence and the correlated high vaccination rate in children in the past few years [29]. Anyway, in our study the most common ADRs related to vaccines were fever, injection-site hypersensitivity and edema, and local vasodilatation (data not shown), that cannot be considered life-threatening, although they led to an ED admission. Thus, considering the high number of vaccine doses administered in Tuscany every year [30], the total number of vaccines-related ADRs detected in the present study is reasonably low and provides a general perception of safety of vaccination in children [31].

The rate of serious ADRs compared to non-serious ones, was lower than observed in other studies, performed in both general and pediatric populations [17, 28]. The lower rate of serious ADRs in this sample may indicate a better quality and safety of prescribing in the setting evaluated. Among the drug classes that deserve particular attention in terms of preventable ADRs, there are “Antinfectives for systemic use” (ATC class J) and “Nervous system medications” (ATC class N). The clinical evaluation of the collected ADR reports identified several cases of misuse and abuse due to accidental drug ingestion for class N. Unintentional exposure among children is an important public health problem [32]. One out of 180 two-year-old child visits an ED for a medication poisoning each year [33], and, during 2010–2011, an average of 1499 children/year aged less than 6 years were evaluated in ED in the United States for unintentional exposure to drugs, including agents of ATC class N [34]. In this context, our evidences underline the importance of careful drug management by parents and caregivers [35], with the final goal of improving the strategies to prevent these kind of adverse events [36].

This study also showed a correlation between the risk of serious ADR and age classes. Children and infants had a significantly lower risk of serious ADRs compared to adolescents, as reported in other studies [37, 38]. To the best of our knowledge, no other publications have previously reported age as a risk factor for serious ADR-related ED admission in children. In general, age-based analyses do not follow a clear pattern and are difficult to evaluate due to the variety of age classifications [10]. Healthcare professionals have to consider important factors related to age, such as pharmacokinetic and pharmacodynamic differences during children growth (i.e., immature tubular function and hypoalbuminemia in neonates, immaturity of blood brain barrier, reduced metabolism and liver function, etc.) [39].

Concerning risk factors associated with ADRs in children, polypharmacy was found as a potential predictor of adverse events. Multiple regression analysis showed a statistically significant correlation between polypharmacy (use of more than 3 drugs) and the risk of presenting to the ED for a serious ADR. Our results are consistent with several published investigations conducted in pediatric patients that also show polypharmacy to be an important factor that predisposes to ADRs [38]. Polypharmacy as a risk factor for ADRs is well characterized in adults and older subjects [40] and descriptive data are also available in children [41], but the risk of serious ADRs had not been addressed previously.

Off-label prescribing has been widely observed in children [42]. However, administration of a drug outside the conditions assessed during clinical trials may result in ADRs [43]. The present study shows no statistically significant difference between on-label and off-label use in terms of ADRs occurrence. Our evidence confirms the data already reported by Palmaro and colleagues on the safety of off-label drug utilization [44].

It is well known that the lack of reliable data on drug safety in the pediatric population is associated with specific issues, among which are the lack of dedicated clinical trials and the non-linear development of pharmacokinetic parameters [45]. Serious ADRs are generally not observed during pediatric clinical trials, especially in case of a latency period before onset [46], and in children as in adults it is not possible to fully investigate the spectrum of serious ADRs prior authorization of most medications [47]. Pharmacovigilance spontaneous reporting systems are subjected to under-reporting of ADRs, including serious ones [48]. Thus, an active pharmacovigilance study like the one presented here represents one of the best strategies to systematically collect, analyze and interpret data on ADRs [17], by means of protocols designed to actively detect ADRs on an ongoing basis within a defined group of people (i.e., children in ED).

The present study has several points of strength. First, we used a computerized monitoring programs and all participants were specifically trained healthcare professionals, who systematically analyzed each single ED clinical record. Furthermore, this is the first retrospective analysis evaluating serious ADRs as cause of ED admission in children over a long period of time in Italy. However, this study has also some limitations. The retrospective nature of our study may have led to an underestimation of the rate of ADR-related ED admissions as a result of missing documented clinical data. Indeed, not all pediatric ADRs were identified, since not all pediatric patients presenting an ADR, even if serious, attend ED. Therefore, the real prevalence of ADR is not known. Last, a control group with no ADRs was not available and this is also a limitation with regard to assessment of causality between ADRs and ED admission.

## Conclusions

Serious ADRs are a relevant clinical event in children and a challenge for pediatricians and health care systems. The present research provided new insights on the factors that might increase the risk of serious pediatric ADRs. Future prospective, large-sample and multicenter studies should focus on other at-risk pediatric settings such as oncology, hematology, neurology, etc. to better understand the impact of ADRs and the effect of programmed preventive actions. In this context, we believe that active surveillance in pharmacovigilance might represent the best strategy to estimate and characterize the clinical burden of ADRs in children, with the final goal of improving the appropriateness of prescribing in the fragile population of pediatric patients.

## Additional files

Additional file 1: **Table S1.** *Most frequent suspect APs among antinfective for systemic use agents (ATC class J) and nervous system medications (ATC class N), overall and stratified according to ADR seriousness.* This table reported the most frequent suspected active principles (APs) among antinfectives for systemic use agents and nervous system medications. (DOCX 48 kb)

Additional file 2: **Table S2.** *Most frequent suspect vaccines, overall and stratified according to seriousness.* This table reported the most frequent suspected vaccines. (DOCX 14 kb)

## Abbreviations

ADR: Adverse Drug Reaction
AP: Active Principles
ATC: Anatomical Therapeutic Chemical
CI: Confidence Interval
DDI: Drug-Drug Interactions
ED: Emergency Department
ROR: Reporting Odds Ratio
SOC: System Organ Class
WHO: World Health Organization
