# Supplementary material for: Characterization of serious adverse drug reactions as cause of emergency department visit in children: a 5-years active pharmacovigilance study
Source: BMC Pharmacol Toxicol. 2018 Apr 16;19:16. doi: 10.1186/s40360-018-0207-4 (PMC5902928; doi:10.1186/s40360-018-0207-4)
Supplement: Supplementary file 1 — Table S1. Most frequent suspect APs among antinfective for systemic use agents (ATC class J) and nervous system medications (ATC class N), overall and stratified according to ADR seriousness. This table reported the most frequent suspected active principles (APs) among antinfectives for systemic use agents and nervous system medications. (DOCX 48 kb) [file 40360_2018_207_MOESM1_ESM.docx]

***Supplementary Table 1:*** *Most frequent suspect APs among antinfective for systemic use agents (ATC class J) and nervous system medications (ATC class N), overall and stratified according to ADR seriousness.*

|  | **Tot**  **drug-ADR pairs**  **N (% out of 289)** | ***Drug-Serious ADR pairs***  ***N (% out of corresponding drug class)*** |
| --- | --- | --- |
| **ATC class J** |  |  |
| Amoxicillin/clavulanate | 149 (51.56) | *29 (19.46)* |
| Amoxicillin | 34 (11.74) | *4 (11.76)* |
| Cefixime | 27 (9.34) | *6 (22.22)* |
| Clarithromycin | 23 (7.96) | *6 (26.09)* |
| Azithromycin | 8 (2.77) | *2 (25.00)* |
| Cefaclor | 7 (2.42) | *5 (71.43)* |
| Ceftriaxone | 7 (2.42) | *3 (42.86)* |
| Cefpodoxime | 5 (1.73) | *1 (20.00)* |
| Ceftazidime | 4 (1.38) | *4 (100.00)* |
| Palivizumab | 3 (1.04) | *0 (0.00)* |
| **ATC class N** |  |  |
| Acetaminophen | 33 (19.64) | *18 (54.55)* |
| Carbamazepine | 20 (11.90) | *9 (45.00)* |
| Lorazepam | 20 (11.90) | *9 (45.00)* |
| Valproic acid | 18 (10.71) | *9 (50.00)* |
| Alprazolam | 6 (3.57) | *6 (100.00)* |
| Lormetazepam | 6 (3.57) | *2 (33.33)* |
| Citalopram | 5 (2.98) | *5 (100.00)* |
| Ethosuximide | 5 (2.98) | *0 (0.00)* |
| Clonazepam | 5 (2.98) | *5 (100.00)* |
| Clomipramine | 4 (2.38) | *0 (0.00)* |
