# Supplementary material for: Characterization of serious adverse drug reactions as cause of emergency department visit in children: a 5-years active pharmacovigilance study
Source: BMC Pharmacol Toxicol. 2018 Apr 16;19:16. doi: 10.1186/s40360-018-0207-4 (PMC5902928; doi:10.1186/s40360-018-0207-4)
Supplement: Supplementary file 2 — Table S2. Most frequent suspect vaccines, overall and stratified according to seriousness. This table reported the most frequent suspected vaccines. (DOCX 14 kb) [file 40360_2018_207_MOESM2_ESM.docx]

**Supplementary Table 2:** Most frequent suspect vaccines, overall and stratified according to seriousness.

|  | **Tot**  **drug-ADR pairs**  **N (% out of 389)** | ***Drug-Serious ADR pairs***  ***N (% out of corresponding drug class)*** |
| --- | --- | --- |
| **Vaccines associated with ADRs** |  |  |
| Meningococcus B, multicomponent vaccine | 67 (17.22) | *22 (32.84)* |
| Diphtheria-hemophilus influenzae B-pertussis-poliomyelitis-tetanus-hepatitis B | 57 (14.65) | *26 (45.61)* |
| Pneumococcus, purified polysaccharides antigen conjugated | 55 (14.14) | *18 (32.73)* |
| Measles, combinations with mumps, rubella and varicella, live attenuated | 52 (13.37) | *1 (1.92)* |
| Measles, combinations with mumps and rubella, live attenuated | 44 (11.31) | *8 (18.18)* |
| Meningococcus C, purified polysaccharides antigen conjugated | 35 (9.00) | *9 (25.71)* |
| Rotavirus, live attenuated | 17 (4.37) | *7 (41.18)* |
| Varicella, live attenuated | 16 (4.11) | *6 (37.50)* |
| Pertussis, inactivated, whole cell, combinations with toxoids | 14 (3.60) | *5 (35.71)* |
| Diphtheria-pertussis-poliomyelitis-tetanus | 10 (2.57) | *2 (20.00)* |
